# Endoparasites of rabbits and hares

**DOI:** 10.1177/10406387241261991

**Published:** 2024-08-06

**Authors:** Katherine Hughes

**Affiliations:** Department of Veterinary Medicine, University of Cambridge, Cambridge, United Kingdom

**Keywords:** *Eimeria*, *Encephalitozoon*, hares, lagomorphs, nematodes, parasites, rabbits

## Abstract

Nematode, cestode, protozoan, microsporidian, and pentastomid parasites affect domesticated and wild rabbits, hares, and jackrabbits of the genera *Brachylagus*, *Lepus*, *Oryctolagus*, *Pentalagus*, and *Sylvilagus*. Some endoparasite infections are of limited or no significance, whereas others have potentially profound consequences. Accurate identification of endoparasites of rabbits, hares, and jackrabbits is an important facet of the work of veterinary pathologists engaged in lagomorph pathology. Here I review endoparasites from the pathologist’s perspective, focusing on pathogenesis, lesions, and implications of infection. Stomach nematodes *Graphidium strigosum* and *Obeliscoides cuniculi* are infrequently pathogenic but may cause gastritis and gastric mucosal thickening. Nematodes *Passalurus ambiguus*, *Protostrongylus* spp., *Trichostrongylus* spp., and *Trichuris* spp. are rarely associated with disease. Adult *Capillaria hepatica* (syn. *Calodium hepaticum*) nematodes and non-embryonated eggs cause granulomatous hepatitis in wild *Oryctolagus cuniculus* and *Lepus europaeus*, resulting in multifocal, off-white, hepatic lesions, which may be misdiagnosed as hepatic eimeriosis. When the rabbit is an intermediate host for carnivore cestodes, the space-occupying effects of *Cysticercus pisiformis* and *Coenurus serialis* may have pathologic consequences. *Eimeria stiedai* is a major cause of white-spotted liver in *O. cuniculus*, particularly in juveniles. Enteric coccidiosis is a noteworthy cause of unthriftiness in young animals, and frequently manifests as diarrhea with grossly appreciable multifocal off-white intestinal lesions. *O. cuniculus* is the natural host for the zoonotic microsporidian *Encephalitozoon cuniculi*. Infection may be acute and focused mainly on the kidneys, or it may follow a chronic disease course, frequently with neurologic lesions. A latent carrier status may also develop.

In the order *Lagomorpha*, family *Leporidae*, domesticated and wild rabbits (genera *Brachylagus*, *Oryctolagus*, *Pentalagus*, and *Sylvilagus*) and hares and jackrabbits (genus *Lepus*) may be affected by nematodes, cestodes, protozoans, microsporidians, and pentastomids. In some cases, endoparasite infection has profound consequences for the host, whereas in other instances, endoparasite infection is of limited or no significance. Accurate diagnosis of endoparasite infections of rabbits, hares, and jackrabbits is thus an important facet of the work of veterinary pathologists engaged with lagomorph pathology.

In this review, I examine identification of endoparasites in lagomorphs from the perspective of the anatomic pathologist, focusing particularly on pathogenesis, lesions, diagnosis, and implications of infection for the infected animal, conspecifics, and other in-contact species. Conduction of fecal egg counts and fecal egg identification will not be discussed in detail as this constitutes part of the specialist work of veterinary parasitologists.

Hares and jackrabbits of the genus *Lepus* spp. will be referred to as hares. The discussion will focus on the European rabbit (*Oryctolagus cuniculus*) and hares, but reference will be made to the pygmy rabbit (*Brachylagus*), Amami rabbit (*Pentalagus*), and various other rabbits and cottontails (*Sylvilagus* spp.), for which there are relevant data.

## Nematodes

### Graphidium strigosum and Obeliscoides spp

Nematodes affecting the stomach of lagomorphs include *Graphidium strigosum* and *Obeliscoides cuniculi. G. strigosum* is particularly associated with infections in the European rabbit,^46^ European brown hare (*Lepus europaeus*),^96^ and Irish hare (*Lepus timidus hibernicus*; an endemic subspecies of the mountain hare).^6^ *O. cuniculi* typically infects European rabbits, snowshoe hares (*Lepus americanus*), and eastern cottontail rabbits (*Sylvilagus floridanus*) in North America.^90,91^ Following the introduction of the eastern cottontail in Italy for hunting purposes, *O. cuniculi* has also been detected in wild European rabbits,^42^ as well as in European brown hares in Ontario, Canada,^90^ suggesting considerable overlap in host range. *Obeliscoides pentalagi* has been described in Amami rabbits (*Pentalagus furnessi*),^49^ and recent descriptions of other disease processes in this species have also recorded the presence of gastric nematodes that were not further described.^74^

Within European rabbits, the context within which the rabbits are kept strongly impacts the prevalence of stomach nematodes. A 1992–1996 survey of wild European rabbits in the United Kingdom found a 78% prevalence of *G. strigosum*,^2^ which supports my own observations examining wild European rabbits from Cambridgeshire, United Kingdom, where I see a high prevalence of infection. By contrast, *G. strigosum* and *O. cuniculi* were detected at low levels in slaughtered European rabbits in Poland, with prevalences of 1.09% and 0.36%, respectively. Stomach nematodes are infrequently reported in European rabbits maintained as pets.^57^

**Figure 1.**
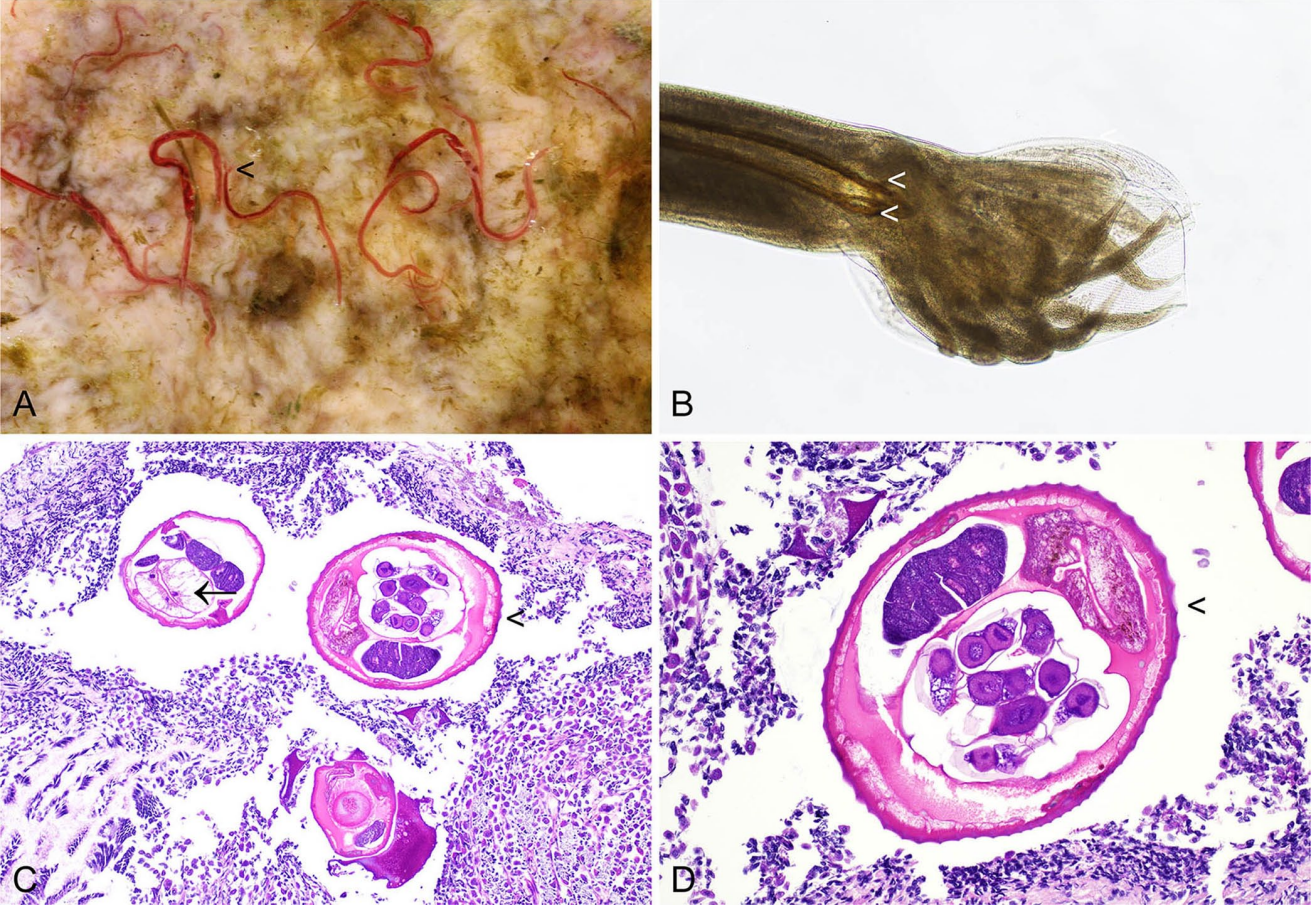
*Graphidium strigosum* in the stomach of a European rabbit. **A.** Red nematodes in close association with the gastric mucosa. The large caudal bursa of males is frequently just visible to the naked eye (arrowhead). **B.** The male has a large caudal bursa and chitinized spicules (arrowheads). **C, D.** Nematodes have a large intestine composed of multinucleate cells (arrow) and a ridged cuticle (arrowheads). H&E.

Both *G. strigosum* and *O. cuniculi* have a direct lifecycle, and third-stage larvae are infective. Infections are apparent macroscopically. In the case of *G. strigosum*, red nematodes are clearly visible in close association with the gastric mucosa (Fig. 1).^40^ The adult males have a prominent caudal bursa that is visible macroscopically.^88^ Microscopically, *G. strigosum* has a ridged cuticle, and the large intestine is lined by multinucleate cells. Brown chitinized spicules may be apparent in sections of adult males (Fig. 1).^51^ Macroscopically, *O. cuniculi* is off-white, and the males also have a prominent bursa and chitinized spicules visible microscopically.^42^ *O. pentalagi* is dark-red.^49^

Gastric nematodes are infrequently pathogenic in rabbits and hares, but *O. cuniculi* may cause gastritis, gastric mucosal thickening, petechiation, and small white mucosal foci. In experimental infections, histologically there may be edema and infiltrates of lymphocytes and eosinophils.^115^ *G. strigosum* infection in European brown hares is suggested to be more pathogenic than in *O. cuniculus*, with high burdens potentially resulting in anemia.^33^ However, this suggestion is somewhat controversial, with another author noting lower *G. strigosum* burdens in hares compared to wild European rabbits sampled from the same locality, and no apparent gastric lesions in the infected hares; the difference in infection level may have been due to relative resistance of hares to the infective third-stage larvae of *G. strigosum*, or different grazing behaviors of hares compared to rabbits may have led to differing levels of exposure.^14^

### Trichostrongylus spp.

Intestinal strongyles include *Trichostrongylus calcaratus* and *T. retortaeformis*, and, in both cases, adults are found in the small intestine. *T. affinis* also affects lagomorphs, and adult nematodes are found in the cecum and large intestine. *T. calcaratus* and *T. affinis* have been detected in eastern cottontail rabbits,^39^ and *T. retortaeformis* is associated with infections in European rabbits and hares.^2,6,14,30^ *T. calcaratus* and *T. affinis* have been identified in eastern cottontail rabbits in Italy and represent nematode species exotic to the Italian ecosystem.^53,135^ Conversely, Italian eastern cottontails may also be infected with *T. retortaeformis*, which naturally occurs in European lagomorphs.^53^

Grossly, *Trichostrongylus* spp. are small and difficult to identify. Histologic features are those of the family *Trichostrongylidae* to which *Trichostrongylus* spp. belong. *Trichostrongylus* spp. receive little attention as a source of significant disease in lagomorphs, although in one study, control European rabbit does had 20% more offspring survive to the point of weaning than did does experimentally infected with *T. retortaeformis*.^34^ Myxoma viral infection may alter the susceptibility of European rabbits to *T. retortaeformis* infection. Rabbits that were infected with myxoma virus had higher nematode burdens and maintained high levels of infection for longer, the latter suggestive of a disrupted ability to clear the nematode infection.^15,22^

### Passalurus ambiguus

*Passalurus ambiguus* is the rabbit pinworm, an oxyurid nematode that is present in the cecum and large intestine. *P. ambiguus* has a direct lifecycle, in which eggs are deposited around the anus and egg ingestion occurs during cecotrophy.

In Finland, *P. ambiguus* eggs were found in 3% of fecal samples from pet rabbits,^86^ and the same proportion of samples were affected in a German study of rabbit fecal specimens.^107^ Prevalence in wild European rabbits is likely to be considerably higher.^2,14^ *P. ambiguus* has also been identified in eastern cottontails,^39,140^ European brown hares,^123^ and the Italian hare (syn. Corsican hare; *L. corsicanus*),^30^ suggesting a wide host range.

**Figure 2.**
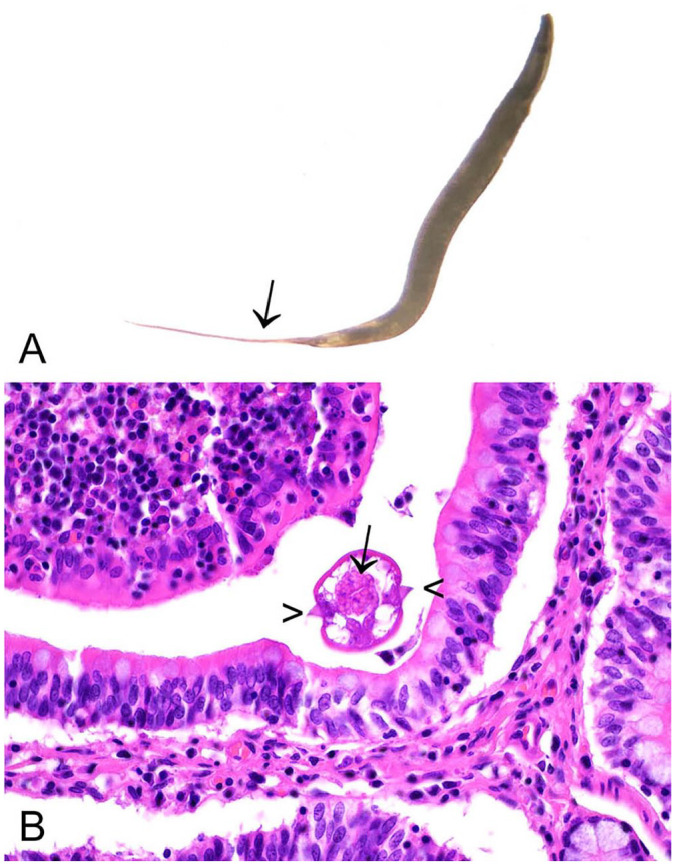
*Passalurus ambiguus* in the cecum of a European rabbit. **A.** Sub-grossly, the females have pointed tails (arrow). **B.** Characteristic histologic features include muscular gastrointestinal tract (arrow), platymyarian musculature and lateral alae (arrowheads). Note, due to the plane of section, the double lateral alae cannot be seen in this image. H&E.

Adult nematodes may be detected in the cecum and large intestine, or in the cecotropes or feces. Nematodes are off-white, thread-like, and 5–10 mm long.^57^ Sub-grossly, the females have pointed tails (Fig. 2A). *P. ambiguus* may also be detected as an incidental finding in histologic sections from the cecum or large intestine. Characteristic histologic features include a muscular esophagus, platymyarian musculature, and double lateral alae (Fig. 2B). *P. ambiguus* infection is typically considered nonpathogenic, but it is possible for heavy infestations in young rabbits to manifest as perianal pruritus, and the presence of large numbers of nematodes may contribute to the enteritis complex.^57^

### Trichuris spp.

*Trichuris* spp. are commonly known as whipworms, and they have a direct lifecycle. Adults are present in the cecum and large intestine. Species infecting lagomorphs include *T. leporis* and *T. sylvilagi. Trichuris* spp. have been identified in wild European rabbits,^45^ eastern cottontails,^39^ European brown hares,^101^ and Amami rabbits.^49^

Macroscopically, *T. leporis* and *T. sylvilagi* are ~20 mm long and have a long, hairlike anterior end that is embedded in the mucosa.^134^ *Trichuris* spp. are aphasmid nematodes; thus, they have bacillary bands, and a distinctive stichosome, which refers to a row of esophageal glands surrounding the esophagus. Eggs have bipolar plugs, as do *Capillaria hepatica* eggs. Like *Trichostrongylus* spp., *Trichuris* spp. infections in rabbits receive little attention and do not appear to be commonly associated with disease. Infections appear to be uncommon in pet animals, with 1 study documenting 1 fecal sample containing *T. leporis* eggs, out of 434 samples,^107^ and another study finding 1 positive sample out of 398.^86^

### Capillaria hepatica (syn. Calodium hepaticum)

As alluded to above, *C. hepatica* (syn. *Calodium hepaticum*) is also an aphasmid nematode. The nematode has a direct lifecycle, a wide host range, and is zoonotic.^13,48,94,109,114^ Embryonated eggs are ingested by natural hosts, and first-stage larvae hatch in the cecum and undergo hepatic migration. In the liver, mature nematodes develop, and fertilized adult females release non-embryonated eggs before dying. Two main mechanisms for continuation of the lifecycle are inferred. Either disintegration of the carcasses of dead hosts facilitates environmental dispersal of non-embryonated eggs, or non-embryonated eggs are consumed by predators and excreted along with the host feces. In favorable conditions, the first-stage larva develops within the egg to an infective stage.^13^

Humans may acquire infection by 2 pathways. Embryonated eggs may be ingested from the environment in a process that is referred to as “true infection.” This is a rare occurrence but is frequently fatal. Conversely, consumption of raw or undercooked livers, and thus non-embryonated eggs, from wild hosts, leads to these eggs passing along the gastrointestinal (GI) tract. The non-embryonated eggs are eventually expelled in the feces, in what is termed a “spurious infection.”^13^

**Figure 3.**
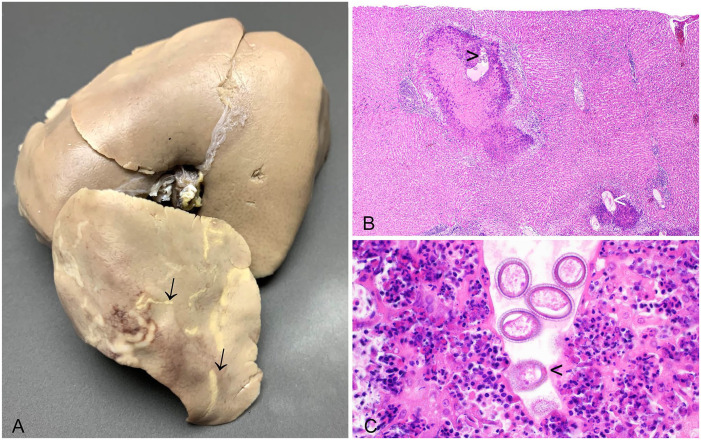
*Capillaria hepatica* in the liver of a European rabbit. **A.** Liver (formalin-fixed) with multifocal linear and serpiginous foci visible on the capsular surface (arrows). **B.** Granulomas with associated nematode eggs (black arrowhead) and degenerate nematode profiles (white arrowhead). H&E. **C.** *C. hepatica* eggs are bi-operculated but opercula (arrowhead) are inconsistently detectable due to plane of section. H&E.

An 8% prevalence of hepatic capillariosis has been reported in wild European rabbits in the United Kingdom.^16^ A 0.9% prevalence of infection has also been reported in European rabbits in La Palma, Canary Islands.^44^ Adult female *C. hepatica* nematodes and non-embryonated eggs cause granulomatous hepatitis, resulting in grossly apparent, multifocal, off-white, hepatic lesions, which may be misdiagnosed macroscopically as hepatic eimeriosis (Fig. 3A).^16^ In histologic sections, the granulomas are distributed randomly, rather than as inflammatory lesions focused on the bile ducts, as in hepatic eimeriosis. Profiles of degenerating nematodes, or more commonly bi-operculated eggs, may be found in the necrotic centers of granulomas (Fig. 3B, 3C). Hepatic capillariosis has also been described in European brown hares^10^ and eastern cottontail rabbits.^79^ As *C. hepatica* infects a wide species range, the role of lagomorphs in its disease ecology merits further contemporary investigation. For example, it is unclear if the 8% prevalence of *C. hepatica* in wild European rabbits is representative of the United Kingdom as a whole or if there are significant regional variations in prevalence. Anatomic pathologists are ideally placed to study this parasite because it will not necessarily be detected using fecal egg counts, and differentiation of the macroscopic liver lesions from those of hepatic eimeriosis is required.^16^

### Protostrongylus spp

*Protostrongylus* spp. are metastrongyle lungworms with an indirect lifecycle. Finnish mountain hares (*Lepus timidus*) and European brown hares examined seasonally from September to the end of February 1998–2001 had a high prevalence of lungworm infection, with 96.5% of mountain hares and 60% of European brown hares exhibiting evidence of infection.^77^ *Protostrongylus* spp. infection has also been reported in a domestic rabbit.^124^

Macroscopically, these metastrongyles may be difficult to identify as they are frequently embedded in lung tissue and are brown and hair-like. In the Finnish study, lesions were most severe in the caudal lung lobes. Macroscopic changes included well-demarcated foci of green and brown pulmonary parenchyma. Histologic lesions comprised mixed inflammation including granulocytes. Lungworm infection did not appear to impact the condition and weight of the hares.^77^

### Baylisascaris spp.

*Baylisascaris* spp. are ascaridoid nematodes.^118^ *B. procyonis* infects common raccoons (*Procyon lotor*) and has a direct lifecycle, although rabbits, hares, and other species can act as paratenic hosts. *B. procyonis* is zoonotic and can cause neurologic disease in humans and wildlife, including eastern cottontails^66^ and European rabbits.^26,119^ Neurologic disease arises due to larval migration (cerebral larval migrans). Histologic lesions comprise malacic migration tracts and variable degrees of encephalitis, granuloma formation, or gliosis. Nematode larvae are inconsistently detected in histologic sections.^26,119^ Raccoon feces are a potential source of infection for domestic rabbits; hence, pets maintained outside, or in proximity to raccoons, are more at risk than house rabbits.^26,81^ The European rabbit has also been experimentally infected with *Ascaris columnaris* (now designated *B. columnaris*).^23^

### *Dirofilaria* spp. and other filarial nematodes

*Dirofilaria* spp. are filarial nematodes. The lifecycle includes a vertebrate definitive host and an arthropod vector, such as a mosquito. Adult female nematodes in the definitive host produce microfilariae that are disseminated in the blood. A feeding arthropod vector ingests the microfilariae. The microfilariae mature in the vector, and the resulting infective larvae migrate to the mosquito’s mouth-parts. From here, the microfilariae enter a new host when the vector feeds. In the new definitive host, the microfilariae mature, migrate to the site where the adult parasites live, and mate. The ensuing microfilaremia continues the lifecycle.^126^

*D. immitis* infection is typically associated with dogs and cats; recorded infections involving lagomorphs are sparse. Experimentally, *D. immitis* infection causes pulmonary nodules in European rabbits.^120^ Two adult *D. immitis* nematodes have been recorded in the lungs of a Japanese hare (*Lepus brachyurus angustidens*). The histologic pulmonary lesions included corrugated, proliferative intimal fibrosis, endoarteritis, and thrombosis.^99^ Similar lesions have been associated with an incidental finding of suspected *D. immitis* in a pet rabbit with spinal lymphoma.^110^

*D. scapiceps* is primarily reported in North American lagomorphs^4,11,73^ but has also been recorded in an individual European brown hare from Macedonia, Greece.^28^ Adult *D. scapiceps* nematodes are found associated with the tendon sheath, most commonly in the tarsal region, or less commonly near the stifle. Adult nematodes can be detected grossly. Histologically, there may be minimal reaction to the presence of the nematodes or there may be a granulomatous response. Microfilariae may also be seen in spaces near adult nematode profiles.^11^

Filarial nematodes have also been detected in 2 adult Iberian hares (syn. Granada hare; *Lepus granatensis*). Sequencing suggested that these filariae were genetically related to *Micipsella numidica*, and they have been provisionally assigned the name *Micipsella iberica* n. sp. The authors of the study highlighted the potential for filarial nematode infections to cause population declines in already fragile lagomorph populations, such as that of the Iberian hare.^32^

### Toxocara spp.

The potential for *T. canis* and *T. cati* to cause visceral larval migrans in rabbits has been demonstrated experimentally.^141^

## Cestodes

The cestodes discussed in this section have a 2-host lifecycle. Adult tapeworms are present in the GI tract of the definitive host. Proglottids containing eggs are passed in the feces of the definitive host, and these are ingested by the intermediate host, which is usually a different species. In the intermediate host, eggs hatch into larvae and encyst in skeletal muscle or other foci (see below). When the intermediate host is consumed by the definitive host, the cysts are digested in the intestine of the definitive host, releasing the scolex, that forms the anterior aspect of the adult cestode, and allowing attachment to the intestinal wall. Development of the adult cestode completes the cycle.^63^ Rabbits and hares may be affected by a range of cestodes, both as an intermediate and definitive host (Table 1).

**Table 1.** Selected cestode parasites of lagomorphs.

Cestode	Lagomorph definitive host	Intermediate host	Reference
*Cittotaenia* spp.	*Oryctolagus cuniculus*	Oribatid (free-living) mites	^2,57,60^
	*Sylvilagus floridanus*		^130,140^
	*Lepus* spp.		^20^
*Ctenotaenia* spp.	*Oryctolagus cuniculus*	Oribatid (free-living) mites	^60^
*Mosgovoyia* spp.	*Lepus europaeus*	Oribatid (free-living) mites	^129^
	*Lepus timidus*		^85,129^
	*Lepus timidus hibernicus*		^6^
*Cysticercus pisiformis* *	NA	*Oryctolagus cuniculus*	^60,112^
		*Sylvilagus floridanus*	^130^
		*Sylvilagus audubonii*	^103^
		*Lepus* spp.	^40^
*Coenurus serialis* †	NA	*Oryctolagus cuniculus*	^2^
		*Lepus* spp.	^9^

NA = not applicable.

* Larval stage of *Taenia pisiformis*.

† Larval stage of *Taenia serialis*.

### Lagomorphs as the cestode definitive host

Theoretically, lesions due to adult cestodes may arise from the physical presence of the cestodes, or due to erosions resulting from the scolex hooks. Hypersensitivity reactions are also theoretically possible.^63^ Rabbits and hares are the definitive hosts for several species of tapeworm, including *Cittotaenia* spp., *Ctenotaenia* spp., and *Mosgovoyia* spp. However, lesions are rarely recorded.

**Figure 4.**
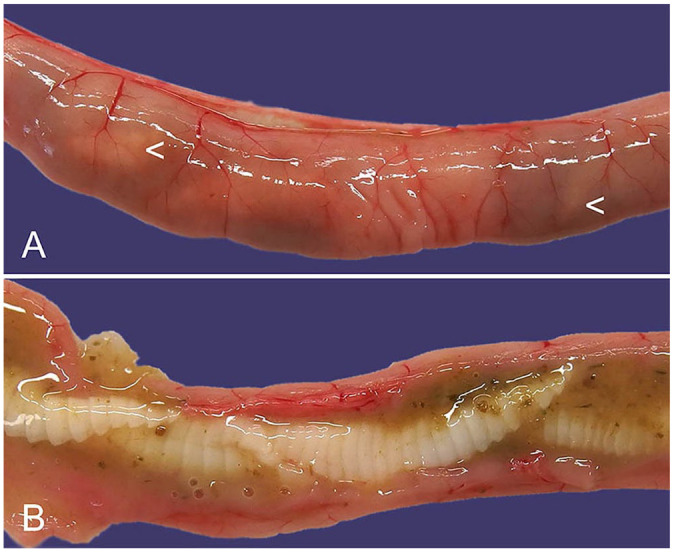
Cestodes in the small intestine of a European rabbit (definitive host). **A.** A cestode is visible through the thin-walled intestine (arrowheads). **B.** Opened intestinal segment from panel A, with an adult cestode, not speciated in this case.

Macroscopic identification of cestode infection is straightforward, with tapeworms frequently visible through the relatively thin-walled small intestine even prior to incision (Fig. 4). Gravid proglottids containing eggs may be noted in the feces.^57^

Myxomatosis virus increases the prevalence and intensity of infection of wild European rabbits infected with *Mosgovoyia pectinata*.^15^ This is similar to the case with *Trichostrongylus* spp.

### Lagomorphs as the cestode intermediate host

**Figure 5.**
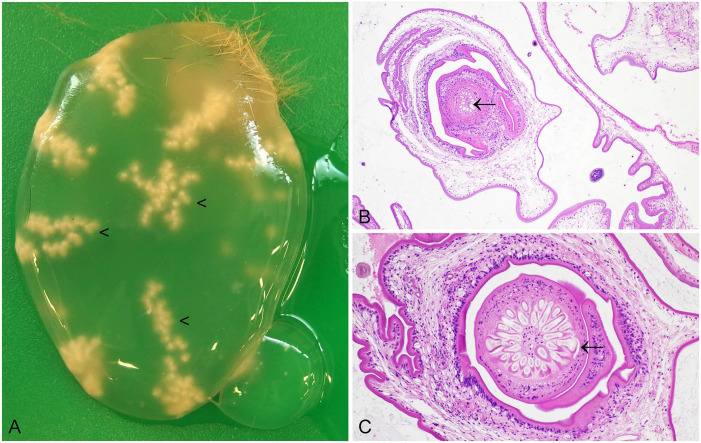
*Coenurus serialis* dissected from skeletal muscle of a European rabbit. **A.** Scolices (arrowheads) are arranged in radiating rows and clusters. The fur present is an artifact. **B.** An invaginated scolex with an armed rostellum (arrow). H&E. **C.** The armed rostellum is composed of hooks (arrow). H&E.

Lagomorphs are an intermediate host for the carnivore cestodes *Taenia pisiformis* and *T. serialis*. A peritoneal location is typical for *Cysticercus pisiformis*,^116^ whereas the larval form of *T. serialis*, *Coenurus serialis*, typically encysts in the musculature.^1^ Cysts are easily identifiable macroscopically and comprise fluctuant structures with translucent contents. *C. pisiformis* tends to form multiple grape-like clusters of cysts, each with a single inverted scolex recognizable as an off-white nodule.^104^ *C. serialis* forms numerous, off-white, ~1-mm nodules, arranged in characteristic radiating clusters and rows (Fig. 5A),^1^ which are the invaginated scolices adhering to the cyst interior. In histologic sections, the armed scolex or scolices of the cestode larval stage may be visualized (Fig. 5B, 5C). Basophilic calcareous corpuscles may also be seen, the function of which is incompletely elucidated. *T. serialis* infection can be zoonotic, with humans acting as an intermediate host.^38^

The space-occupying effects of *Cysticercus pisiformis* and *Coenurus serialis* may have location-dependent pathologic consequences for the lagomorph. For example, exophthalmos has been reported as a consequence of a *T. serialis* coenurus located in the conjunctival fornix of a pet dwarf lop rabbit^97^; facial swelling and reduced appetite were reported in a pet lionhead rabbit with mandibular *C. serialis*.^106^ Although *C. serialis* typically encysts in the musculature in rabbits, GI stasis has also been reported due to the atypical presence of a mid-abdominal *C. serialis* cyst.^1^

The migration of *T. pisiformis* larvae in eastern cottontail rabbits can cause hepatic inflammation and necrosis.^116^ Similarly, granulomatous hepatitis has been recorded in European rabbits associated with numerous *T. pisiformis* cysticerci of various developmental stages.^54,117^

## Coccidia

Coccidia are intracellular obligatory parasites in the phylum *Apicomplexa*. Some are monoxenous (utilizing a single host species), such as *Eimeria* spp. in most instances, whereas other species are heteroxenous (use 2 hosts).

### Eimeria stiedai

*Eimeria stiedai* infects biliary epithelium and is a major cause of white-spotted liver in European rabbits. In wild rabbits in the United Kingdom, white-spotted liver caused by *E. stiedai* is associated with the juvenile age class.^16^ *E. stiedai* is a common pathogen of young farmed rabbits.^40^ Sporadic, clinically significant, infections in pet rabbits are documented.^95^ Cottontail rabbits do not appear to develop natural infections of *E. stiedai*, but may be infected experimentally.^62^

*E. stiedai* transmission is fecal-oral. Unsporulated *Eimeria* oocysts pass into the GI tract and are shed in the feces. Sporulation occurs within 1–4 d, and results in the oocysts becoming infective. Oocysts are resistant to a range of environmental conditions. Sporulated oocysts may be ingested via contaminated food or water. Auto-infection through cecotrophy does not occur because cecotropes contain unsporulated *Eimeria* oocysts.^127^ Schizogony is the phase of asexual reproduction, and gametogony is the phase of sexual reproduction that leads to oocyst formation, and these occur in biliary epithelial cells.

**Figure 6.**
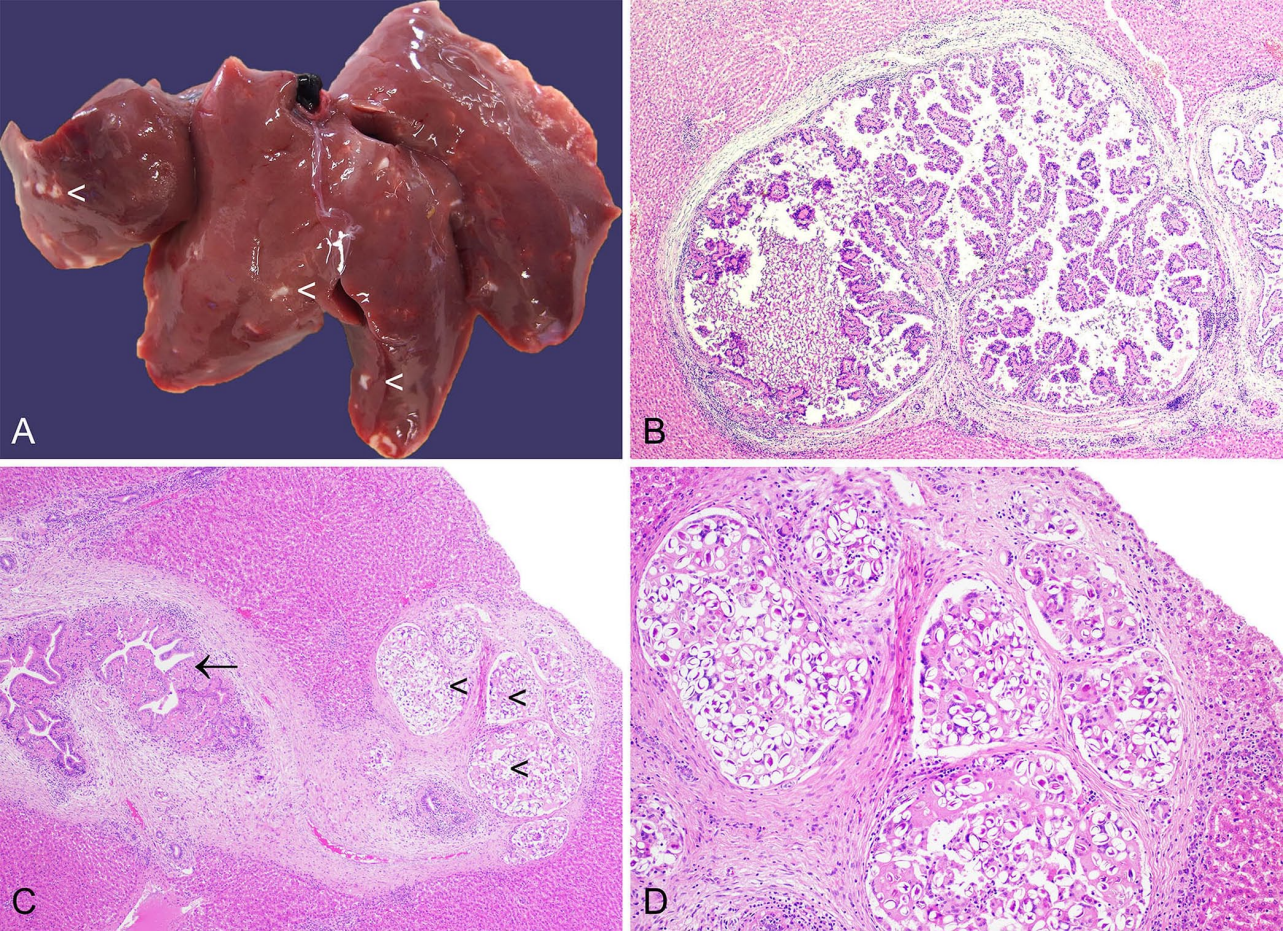
*Eimeria stiedai* in the liver of a European rabbit. **A.** Liver with numerous off-white bosselated foci (arrowheads). **B.** Bile ducts are expanded by arborizing fronds of biliary epithelial cells, many of which are expanded by *E. stiedai* macrogamonts and oocysts. H&E. **C.** Bile ducts are surrounded by fibrosis (arrow). Large numbers of free oocysts are present (arrowheads). H&E. **D.** Oocysts are surrounded by granulomatous inflammation and fibrosis. H&E.

*E. stiedai* infections result in off-white, frequently bosselated, hepatic foci (Fig. 6A). We devised a grading scheme to delineate the extent of the lesions.^16^ Although there is a high prevalence of infection in wild rabbits, most animals have mild gross lesions that are likely not clinically significant.^16^ Histologically, various developmental stages of the parasite, including meronts (merozoites), macro- and microgamonts, and oocysts may be observed in the biliary epithelium, with destruction of parasitized cells, and in the bile duct lumen. Lesions are frequently accompanied by periductular fibrosis, infiltration of inflammatory cells, including macrophages and heterophils, and bile duct hyperplasia (Fig. 6).^7,16,133^

Diagnosis is readily achieved by a combination of gross and microscopic examination. Infection with *C. hepatica* constitutes a differential diagnosis for the gross lesions, and a number of other potential or reported causes of white-spotted liver should also be considered, including *Cysticercus pisiformis*, and bacteria including *Yersinia pseudotuberculosis* and *Francisella tularensis*.^16^ Impression smears have been used to aid in the diagnosis of hepatic eimeriosis.^5^

### Enteric *Eimeria* spp

Many species of *Eimeria*, of varying pathogenicity, infect enterocytes. The lifecycle is as described for *E. stiedai* but with infection of enterocytes rather than biliary epithelial cells. Level of pathogenicity and localization in the intestine vary and have been reviewed.^36,100^ Although their sites of development within the intestine overlap, it is likely that each species of *Eimeria* has its own niche within the intestinal microenvironment.^100^

Authors vary slightly in their classification of *Eimeria* spp. as high, intermediate, or low pathogenicity, but in general, for European rabbits, *E. flavescens*, *E. intestinalis*, *E. irresidua*, *E. magna*, and *E. piriformis* are considered of “high” or “intermediate” pathogenicity, and some authors also include *E. media* as “mildly pathogenic” or “pathogenic.”^100^ *E. brachylagia* has been described in the pygmy rabbit. *E. brachylagia* has been suggested to be potentially extremely pathogenic, with the caveat that the affected animals may have experienced stress due to captivity.^37^ *E. furnessi* n. sp., *E. hilleri* n. sp., and *E. sagentae* n. sp. have been identified in fecal samples from the Amami rabbit. Further research is required to definitively establish their pathogenicity.^136^ Of the 32 species of hares, only 10 have been examined for coccidia and, from these species, 43 *Eimeria* spp. have been identified, with some overlap with the domestic rabbit and cottontail rabbits.^35^ Thus, across genera of rabbits and hares, a plethora of enteric *Eimeria* spp. exist and are of varying clinical significance. However, some general patterns of pathology emerge across lagomorphs infected with pathogenic *Eimeria* spp.

Enteric coccidiosis causes unthriftiness and diarrhea in young, recently weaned animals. This can be a particular issue in breeding units, primarily affecting European rabbits, but also where hares are kept in captivity.^21,142^ Young weanling wild cottontail rabbits that are presented to wildlife rehabilitation centers may also have enteric eimeriosis. This manifests as diarrhea, potentially with additional GI stasis and cecal impaction.^102^ Enteric coccidiosis is also a major cause of mortality in young, wild European brown hares.^41^

**Figure 7.**
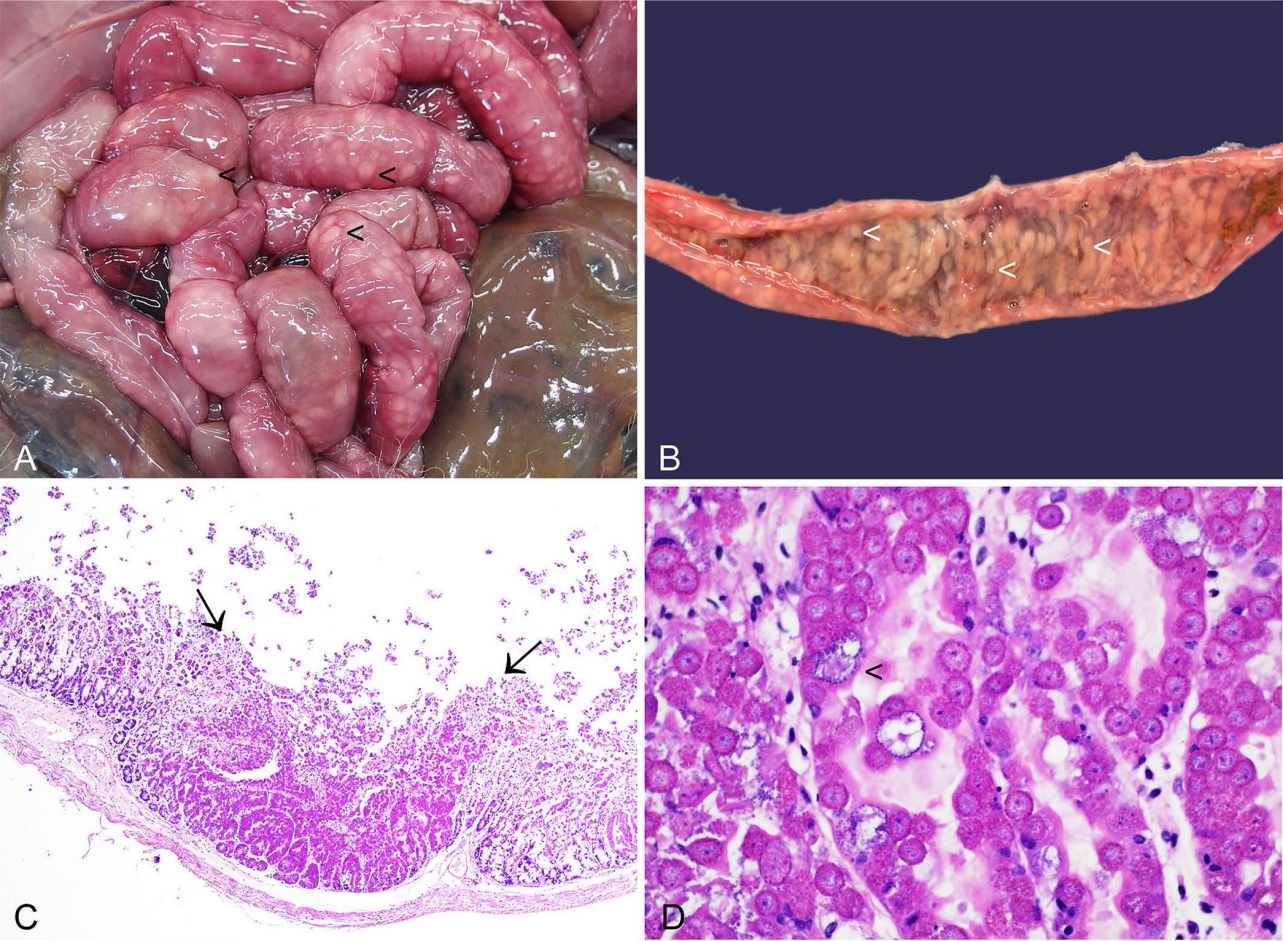
Enteric coccidiosis in the small intestine of a European brown hare. **A.** Prominent off-white nodules are visible on the serosal surface of the small intestines (arrowheads). **B.** The off-white nodules are also visible on the mucosal surface (arrowheads). **C.** In sub-grossly apparent foci (delineated by arrows), corresponding to the off-white nodules seen grossly, most of the enterocytes of the small intestine are expanded and effaced by *Eimeria* spp. H&E. **D.** *Eimeri*a spp. macro- and microgametes (arrowhead) replace the enterocytes. H&E.

Infected juveniles may have low body condition score and extensive fecal soiling in perianal foci and affecting the hindlimbs. High burdens of enteric *Eimeri*a spp. manifest as numerous white nodules expanding the intestinal wall and grossly visible from both serosal and mucosal aspects (Fig. 7A, 7B). Various developmental stages of coccidia are microscopically detectable within enterocytes (Fig. 7C, 7D), together with necrosis and inflammation.^102,104^ Fecal examination provides quantification of fecal oocyst numbers and is therefore a valuable adjunct to diagnosis. However, it is important to note that oocysts can be detected in the feces of clinically normal animals because not all *Eimeria* spp. are pathogenic. In addition, the *Eimeria* asexual reproduction phase can lead to necrosis and inflammation prior to shedding of oocysts.^102^ Fecal oocyst counts should therefore be interpreted in parallel with gross and microscopic findings.

Intussusception has been reported in an adult female European brown hare with high levels of *E. leporis* infection; the intussusception may have resulted from intestinal hypermotility due to high levels of endoparasitism.^89^

### Cryptosporidium spp.

*Cryptosporidium* spp. can infect intestinal and/or respiratory epithelial cells. A study including modest numbers of European rabbits and European brown hares identified *C. parvum* in feces from 7% of wild rabbits in central mainland Britain, but in neither of 2 hares.^131^ Fecal positivity has also been documented in wild Iberian hares from southern Spain.^111^ Cryptosporidia have been visualized histologically in a wild juvenile female eastern cottontail rabbit found dead in Illinois.^116^

As in other species, cryptosporidiosis is predominantly of clinical consequence in younger rabbits and is more likely to constitute a clinical problem in facilities in which rabbits are reared intensively.^71,125^ Infection with *Cryptosporidium* spp. may contribute to a multifactorial syndrome of enteritis.^57^ Histologic diagnosis is based on the presence of apical, weakly basophilic, ~2-µm, round protozoa that are present within a parasitophorous vacuole.^116^ Villus atrophy may be observed.

### Toxoplasma gondii

*Toxoplasma gondii* is a heteroxenous apicomplexan parasite. Sexual reproduction occurs in the intestine of the definitive host, a felid. Infection of intermediate hosts is by ingestion of food or water contaminated with oocysts from the definitive host. Asexual reproduction occurs in tissue cysts of intermediate hosts. Intermediate hosts are birds and mammals, including rabbits and hares. The carnivorous definitive hosts are infected by consumption of tissue cysts in the intermediate hosts.^3,132^ Infection may also occur in utero or via milk.

Toxoplasmosis has been a noted cause of mortality in hares.^56,70,105,121^ In contrast, lesions associated with toxoplasmosis are rarely diagnosed in European rabbits.^31^ Antibodies to *T. gondii* have been detected in eastern cottontail rabbits from Missouri and Kansas.^128^ The apparent variable level of susceptibility to clinical infection of the hares compared to European rabbits and cottontail rabbits merits further investigation.^3^

Hares experimentally infected with *T. gondii* oocysts develop hemorrhagic enteritis, mesenteric lymphadenopathy with hyperemia, splenomegaly, and necrotic hepatic foci.^121^ Diagnosis of toxoplasmosis is usually achieved via histopathology. Necrotic lesions in multiple organ systems are associated with protozoal tachyzoites or bradyzoite cysts and variable levels of attendant inflammation.^31,121^ Immunohistochemistry (IHC) may aid diagnosis.^31^

*T. gondii* is zoonotic. Rabbits and hares are hunted for meat for human consumption in some countries, which has potential implications for zoonotic disease transmission.^3,55^ Consumption of undercooked meats, including game, is an established risk factor for toxoplasmosis in pregnant women.^24^

### Sarcocystis spp.

*Sarcocystis* spp. are also heteroxenous. Rabbits and hares act as intermediate hosts, and *Sarcocystis* spp. bradyzoite cysts can be found in skeletal and cardiac muscles. *S. cuniculi* has been detected in European rabbits,^19^ and *S. leporum* in cottontail rabbits.^43^ In both cases, the definitive host is the cat. Prevalence appears regionally variable.^55^ *Sarcocystis* spp. cysts have also been documented in European brown hares.^98^

**Figures 8, 9.**
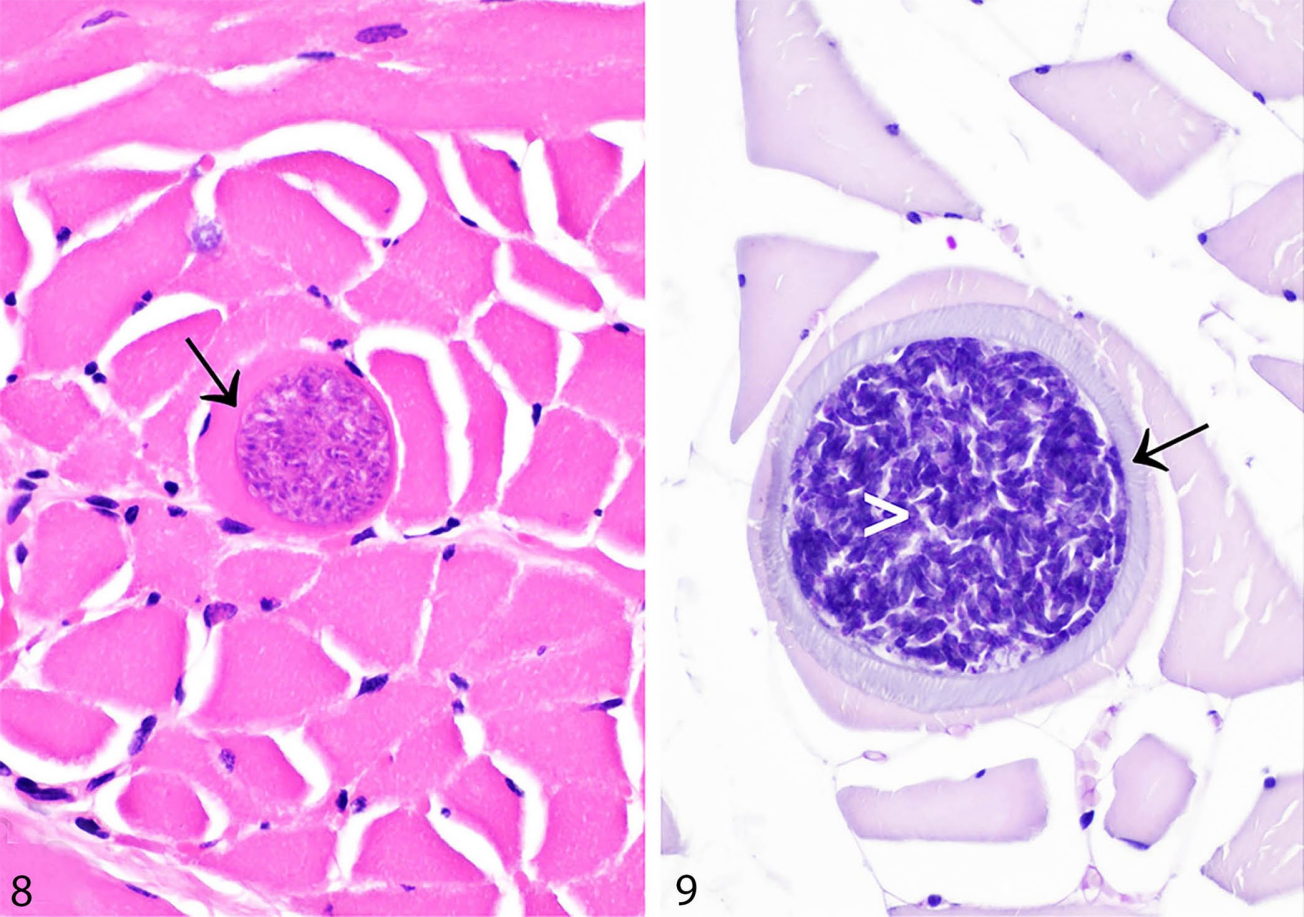
*Sarcocystis* spp. in skeletal muscles of rabbits and hares. **Figure 8.** Rare myocytes in the tongue of a European brown hare are expanded by *Sarcocystis* spp. cysts. The myocyte sarcoplasm is peripheralized by the cyst (arrow). H&E. **Figure 9.** The wall of a *Sarcocystis* spp. cyst in the hindlimb of a European rabbit is apparent (arrow), and the cyst contains numerous PAS-positive zoites (white arrowhead). PAS.

Mild infections are usually undetectable grossly, but severely parasitized muscles may have macroscopically visible streaks. Histologically, sarcocysts are round to elongate-oval and ~50 µm in diameter, with a thin hyalinized wall. They contain abundant crescent-shaped zoites each measuring ~4 × 10–15 µm (Figs. 8, 9).^122^ It has been suggested that adiaspiromycosis could be misdiagnosed as protozoal cysts.^50^ We have identified adiaspores in rabbit lung and lymph node as either an incidental or potentially clinically relevant finding. The bi- or trilaminar wall of adiaspores, together with the granular-to-foamy, basophilic core and location, aid in distinction from *Sarcocystis* spp. cysts.^64,65^

Humans infected with non-human *Sarcocystis* spp. (e.g., through consumption of undercooked game) can become aberrant intermediate hosts, or so-called “dead-end hosts,” with possible attendant clinical signs. However, the potential for this in the case of consumption of rabbit and hare *Sarcocystis* spp. cysts is, to my knowledge, poorly characterized.^55^

### Other protozoa

European rabbits may be infected with *Trypanosoma nabiasi*, a hemoflagellate protozoan. It is believed to have minimal effects on most hosts.^93^ *Hepatozoon* spp. have been detected in 1 of 171 (0.6%) European brown hares from Croatia.^137^ The flagellate *Giardia duodenalis* has been identified in wild, farmed, and pet European rabbits and wild Iberian hares.^8,68,82,111^

## Microsporidia

Microsporidia are basic eukaryotic organisms that are obligate intracellular parasites. Their cellular structure is characterized by an absence of typical mitochondria, Golgi apparatus, and peroxisomes, and the presence of small, prokaryote-like, ribosomes. Microsporidia are phylogenetically closely related to fungi.^84^

### Encephalitozoon cuniculi

The European rabbit is the natural host for the zoonotic microsporidian parasite *E. cuniculi*, which is present worldwide and has a wide host range. The lifecycle is direct, with both horizontal and vertical (transplacental) transmission occurring in rabbits.^84,138^

In rabbits, postnatal infection usually occurs within 6 wk following transmission from an infected dam or other infected animal. Transmission is via ingestion or inhalation of spores that are present in the urine of infected rabbits in large numbers for 1–2 mo following infection. Spores are relatively resistant to environmental conditions, with survival times outside the host of up to 6 wk. By 3 mo post-infection, most spore dissemination is complete, although there may be future intermittent periods of shedding.^76^

*E. cuniculi* spores infect cells via a polar tube or filament that extrudes during germination. Initial target organs for infection are highly vascularized organs, such as the lungs, liver, and kidney.^76^ Nervous system involvement may occur at a more advanced disease stage, with a historic study demonstrating that brain lesions occur at least 8 wk after a positive antibody titer.^25^ However, other authors detected *E. cuniculi* in the medulla oblongata, cerebellum, and leptomeninges at 2 wk post-infection.^67^ These 2 findings are not necessarily contradictory as the latter study detected the organism using IHC, whereas the earlier study assessed lesions that would postdate the presence of organisms.

Once within a host cell, the infective sporoplasm forms a parasitophorous vacuole. It then matures into meronts that undergo an asexual reproduction phase called merogony. In a stepwise process, meronts differentiate into sporonts, and subsequently mature spores. This process is termed sporogony. Spores have a resistant spore wall and develop a polar tube. Eventually, when engorged with spores, the parasitophorous vacuole ruptures to release the spores, which can then disseminate locally and to more distant sites via the vasculature.^76,84^ In an immunocompetent rabbit, cell rupture is associated with an inflammatory response, and thus chronic, subclinical infections can ensue.^76^

Serologic studies of healthy pet rabbits suggest that there may be relatively wide contact with the pathogen, with seroprevalence of 23–52% of animals in the United Kingdom, and 68% of animals in Italy.^29,59,72^ In a German study, 18% of animals with no clinical signs detected upon physical and neurologic examination were positive for *E. cuniculi* antibodies, with an equivalent figure of 48% for rabbits with clinical signs of disease.^61^ Although it might be tempting to ascribe a role in the dissemination of infection to wild rabbits, we found that most of a cohort of U.K. wild rabbits did not have renal lesions consistent with *E. cuniculi infection.*^78^ Similarly, a different U.K. population of wild rabbits did not have *E. cuniculi* antibodies.^18^ This trend is seen in other countries as well. A cross-sectional assessment of wild European rabbits and Iberian hares from southern Spain did not detect *E. cuniculi* by PCR,^87^ and wild lagomorphs examined from northern Spain did not have lesions consistent with encephalitozoonosis.^40^ It is therefore important to appreciate that *E. cuniculi* has a broad host range and that other species may be important in infection dynamics.^75,78,84,92^

The pathology picture in cases of rabbits with encephalitozoonosis may be variable. Patients may be presented with polyuria and polydipsia, poor body condition, and inappetence, and have severe renal disease secondary to infection with the organism. In this case, the kidneys may appear macroscopically multifocally red and irregularly pitted, or may have minimal gross changes. Granulomatous or pyogranulomatous interstitial nephritis may be observed histologically. Organisms are small, round-to-oval, amphophilic-to-basophilic structures on H&E. Organisms may be detected in the tubular lumina, or in the surrounding interstitium. On H&E, organisms may be variably distinct, and detection of spores may be enhanced by use of additional histochemical stains. Chronic interstitial nephritis, together with renal fibrosis causing macroscopic sub-capsular indentations, may also be detected at autopsy.^108^ These more chronic cases may also have attendant histologic neurologic lesions, and animals may become latent carriers.

**Figure 10.**
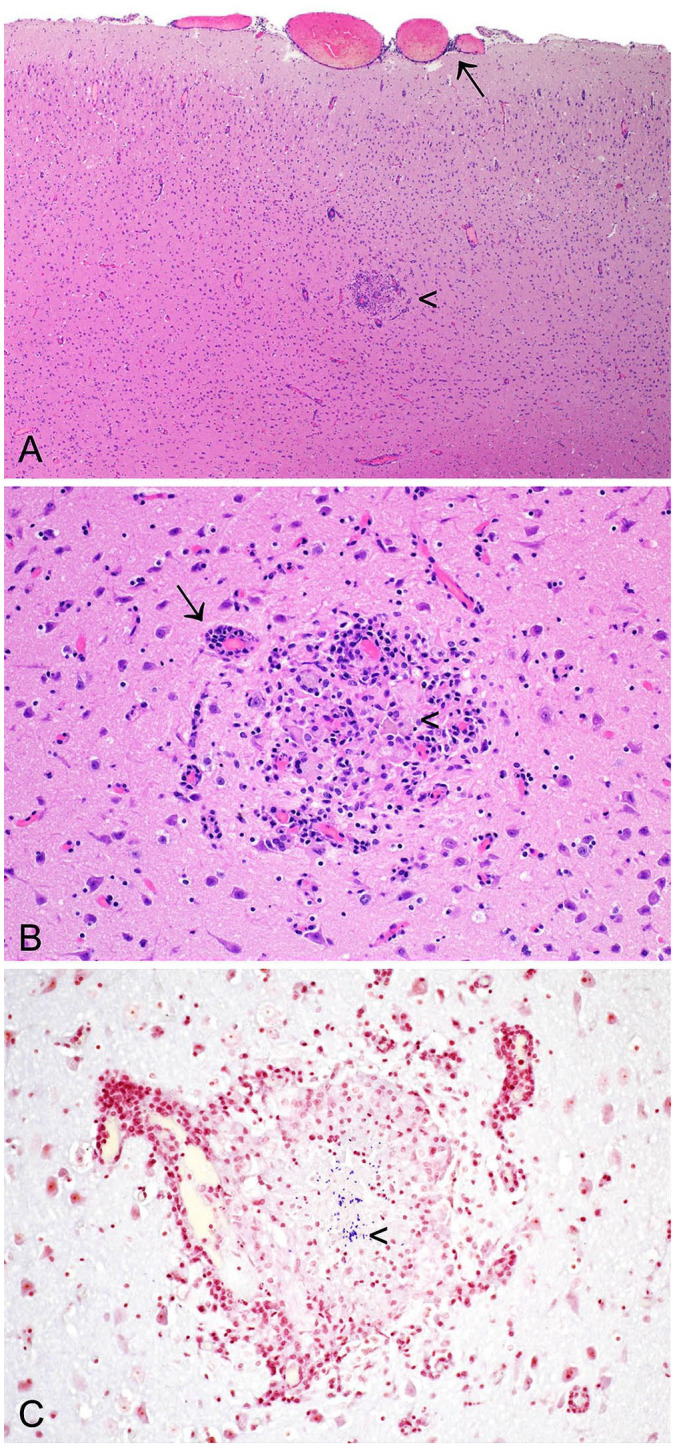
*Encephalitozoon cuniculi* in the cerebrum of a European rabbit. **A.** Lymphocytes and plasma cells multifocally expand the meninges, particularly in perivascular foci (arrow). A granuloma is present in the gray matter (arrowhead). H&E. **B.** There is perivascular cuffing (arrow), and a granuloma (arrowhead) is composed of loosely arranged macrophages, with abundant cytoplasm, and lymphocytes. H&E. **C.** Gram-positive microsporidian spores (arrowhead) are present within the granuloma. Gram stain.

Rabbits with neurologic lesions may have a history of subtle behavior change, a minor head tilt, or severe vestibular disease.^58^ Lesions observed in the CNS may include perivascular cuffing, meningitis, and multifocal granulomatous encephalitis, with organisms visible (Fig. 10).^25,80^ A 6-point grading scheme has been described for brain lesions associated with encephalitozoonosis, reflecting the apparent level of organization and chronicity of the lesion.^80^ In that study, the most frequently observed lesion was the type 4 lesion, comprising a necrotic center, with epithelioid macrophages, gemistocytic astrocytes, occasional multinucleate giant cells, and less prominent lymphocyte infiltration.^80^ In rabbits infected with *E. cuniculi*, granulomatous infiltrates may also be observed in the liver, lungs, and heart.^80^

Encephalitozoonosis may cause intraocular lesions, including cataracts, uveitis, and hypopyon. Intraocular lesions are typically unilateral. The pathogenesis is considered to be in utero (vertical) infection, with subsequent sporogony leading to lens rupture, and ensuing phacoclastic uveitis.^58^ In these cases, spores may be identified in lens epithelial cells and macrophages using IHC.^52^

Although the clinical history, and macro- and microscopic findings, may lead to a strong suspicion of encephalitozoonosis, spores may be subtle and hard to detect in H&E-stained sections. This difficulty is exacerbated by the presence of necrosis and inflammation. In a study comparing 14 histochemical stains for the detection of microsporidian spores, it was concluded that modified trichrome stain and Gram stain were optimal for use with light microscopy (Fig. 10C), and calcofluor white stain was beneficial for detection of spores by ultraviolet light microscopy.^113^ IHC, if available, is likely to be more sensitive than histology for the detection of spores, and real-time PCR is likely even more sensitive.^19,80^ *T. gondii* is a differential for *E. cuniculi* histologically.^19^ The morphology of *T. gondii* cysts and tachyzoites distinguishes them from the spores of *E. cuniculi*.

Although most consideration of *E. cuniculi* infection in rabbits and hares has focused on infection in European rabbits, PCR has demonstrated *E. cuniculi* DNA in eastern cottontails in northern Italy.^143^

### Encephalitozoon intestinalis

*Encephalitozoon intestinalis* (formerly *Septata intestinalis*) is the second most common human microsporidian and primarily infects enterocytes, but may also infect macrophages, fibroblasts, and endothelial cells.^17,139^ It causes diarrhea in both immunocompetent and immunocompromised hosts. *E. intestinalis* DNA has been amplified, by nested PCR, from 2.7% of fecal samples from rabbits from pet shops in Sichuan province, China.^27^ Another study that also examined fecal samples, this time from wild European rabbits and Iberian hares from the Autonomous Region of Andalusia in southern Spain, detected DNA from *E. intestinalis* in 1 of 438 rabbits examined.^111^ The microsporidian has also been documented in kidney from 1 wild rabbit and in 2 brains and 1 kidney from 3 wild Iberian hares, from southern Spain. The latter study did not include samples from the GI tract as it was focused on potential zoonotic transmission through consumption of organs from lagomorphs.^87^

In future studies, it would be informative to examine histologic sections from positive lagomorphs, to attempt to correlate disease-associated lesions with amplification of microsporidian DNA.

### Enterocytozoon bieneusi

*Enterocytozoon bieneusi* can infect enterocytes, biliary, pancreatic, and respiratory epithelial cells, as well as macrophages.^139^ It is the most frequently diagnosed microsporidian infection in humans and is linked to cases of diarrhea that may be persistent in immunocompromised hosts.^83^ Transmission is via the fecal-oral route. Although *E. bieneusi* DNA was not detected in fecal samples from wild European rabbits and Iberian hares from southern Spain,^111^ *E. bieneusi* DNA was detected in kidney samples from 3 wild European rabbits sampled from the same region.^87^ *E. bieneusi* DNA has also been identified in 15.4% of fecal samples from rabbits from pet shops in Sichuan province, China.^27^ As with *E. intestinalis*, correlation of microsporidian genetic material with any lesions in target organs would aid in assessment of the significance of these findings.

## Pentastomes

Class *Pentastomida* are arthropods. The adults reside in vertebrate upper respiratory passages and resemble segmented worms.

### Linguatula serrata

*Linguatula serrata* adults infect the nasal cavities and sinuses of domestic carnivores and foxes. Adults are 1.5–2.0 cm long (males) to 8.5 cm long (females), tongue-shaped, and have transverse striations. Eggs are disseminated from the host via nasal secretions, or are swallowed and expelled in the feces. Herbivores, including rabbits and hares, can become infected via ingestion of eggs. In the intermediate host, larvae migrate through the intestinal wall and encyst in the viscera. Several molts ensue before the formation of infective nymphs that can infect the definitive host. In the carnivore definitive host, the nymphs migrate to the nasal cavity and mature into adult pentastomes. Disease is zoonotic and humans can be infected as either intermediate or definitive hosts.^47^

*L. serrata* larvae were detected in 16 of 84 (19%) European brown hares from Macedonia, Greece, where they were present on the GI tract serosal surface.^28^ Nymphs have also been characterized from European rabbits from Australia^12^ and European brown hares from Romania.^69^ Although pathology descriptions are somewhat limited, it appears that nymphs present in the lung may elicit a granulomatous response.^69^ Research on *L. serrata* in wildlife is limited but is vital to understanding the disease ecology of this parasite.^47^

## Future perspectives

Accurate gross and microscopic identification of endoparasites in rabbits and hares is an important element of the diagnostic work of veterinary anatomic pathologists. There are also abundant opportunities for pathologists to contribute to research in this field. For example, studies detecting microsporidia in wild lagomorphs have capitalized on the availability of molecular techniques to identify microsporidian genetic material, but there is a need to clarify whether microsporidia such as *E. intestinalis* and *E. bieneusi* cause disease in these hosts. Furthermore, given the zoonotic nature of rabbit and hare endoparasites, such as *C. hepatica*, *E. cuniculi*, and others, veterinary pathologists are ideally poised to make valuable contributions to the understanding of the disease ecology of these pathogens in the context of a One Health research program.
